# Cerebral proton magnetic resonance spectroscopy demonstrates reversibility of N-acetylaspartate/creatine in gray matter after delayed encephalopathy due to carbon monoxide intoxication: a case report

**DOI:** 10.1186/1752-1947-8-211

**Published:** 2014-06-19

**Authors:** Marco Bo Hansen, Daniel Kondziella, Else Rubæk Danielsen, Vibeke Andree Larsen, Erik Christian Jansen, Ole Hyldegaard

**Affiliations:** 1 Hyperbaric Oxygen Treatment Unit, Department of Anesthesia, Centre for Head and Orthopedics, Copenhagen University Hospital, Rigshospitalet, Blegdamsvej 9, Copenhagen DK-2100, Denmark; 2 Department of Neurology, Copenhagen University Hospital, Rigshospitalet, Blegdamsvej 9, Copenhagen DK-2100, Denmark; 3 Department of Radiology, Diagnostic Centre, Copenhagen University Hospital, Rigshospitalet, Blegdamsvej 9, Copenhagen DK-2100, Denmark

**Keywords:** CO poisoning, Magnetic resonance spectroscopy, Magnetic resonance imaging, Encephalopathy, Choline, N-acetylaspartate

## Abstract

**Introduction:**

Predictive markers for long-term outcome in carbon monoxide-intoxicated patients with late encephalopathy are desired. Here we present the first data demonstrating a full reversibility pattern of specific brain substances measured by cerebral proton magnetic resonance spectroscopy in a carbon monoxide-intoxicated victim. This may provide clinicians with important information when estimating patient outcome.

**Case presentation:**

We report the case of a 40-year-old Caucasian woman with severe carbon monoxide poisoning who was treated with five repetitive sessions of hyperbaric oxygen therapy in a multiplace chamber (100 percent oxygen with a ventilator, 90 minutes exposure to 2.8 atmospheres absolute). Initially, our patient recovered completely after three days of hospitalization, but became encephalopathic after a lucid interval of four weeks. An examination of the brain with cerebral proton magnetic resonance spectroscopy showed a dramatically decrease in N-acetylaspartate to total creatine ratios and elevated lactate levels in the gray matter. Subsequently, our patient received six additional sessions of hyperbaric oxygen therapy with only minimal recovery. At six-month follow-up our patient showed significant improvement in cognition and neuromuscular coordination. Extraordinarily, the cerebral proton magnetic resonance spectroscopy measurements at relapse compared to measurements at follow-up (217 days post insult) revealed full reversal of the severe abnormalities in mid-occipital gray matter and partial reversal in white matter.

**Conclusions:**

The present case indicates that cerebral proton magnetic spectroscopy provides valuable information on brain metabolism in patients presenting with delayed encephalopathy after acute carbon monoxide intoxication. The full reversal of N-acetylaspartate to total creatine ratios in gray matter has, to our knowledge, never been described before and shows that severe, initial measurements may not predict poor long-term patient outcome.

## Introduction

Carbon monoxide (CO) is a nonirritant gas that remains a leading cause of poison-related death worldwide [1]. Acute low-dose CO intoxication seems to cause reversible neuropsychological impairment [2] whereas patients suffering from high-dose exposure may show a complex clinical pattern. In the latter case, delayed encephalopathy and neuropsychiatric sequelae may occur after a lucid interval of three days to four weeks after the initial recovery from the acute stage [3]. Although neuropsychiatric symptoms are common, delayed encephalopathy is a rare and poorly explained complication. However, the condition is important in terms of outcome in this group of patients. At present, there is no effective treatment for delayed encephalopathy and the precise pathophysiological mechanisms need to be elucidated.

Magnetic resonance imaging (MRI) is routinely used to depict abnormalities in the CO-intoxicated brain despite some difficulties with the precise interpretation of hyperintense areas on T2-weighted images as it may represent various histological changes [4]. Additionally, lesions in the globus pallidus are often considered to be a pathognomonic sign for CO intoxication, but MRI scans are sometimes within normal limits despite clinical and neuropsychological impairment [5].

Cerebral proton magnetic resonance spectroscopy (MRS) provides valuable information on brain function in CO-intoxicated patients that cannot be seen on MRI. It is a noninvasive method that allows the clinician to monitor the neurochemical substances in the brain, which gives new insight into the pathophysiology of CO intoxication and may help the clinician to estimate the risk of chronic deficits after acute CO poisoning [6]. In particular, predictive markers for late encephalopathy are desired in order to allow appropriate triage of the patients for hyperbaric oxygen therapy (HBOT). However, the literature regarding MRS as a diagnostic and prognostic tool after CO poisoning is sparse [6-9].

To provide additional information on the effects of CO on the central nervous system and its correlation to MRS, we report the case of a patient with delayed encephalopathy after acute CO poisoning showing an unusual reversibility pattern when measured by MRS.

## Case presentation

**Figure 1:**
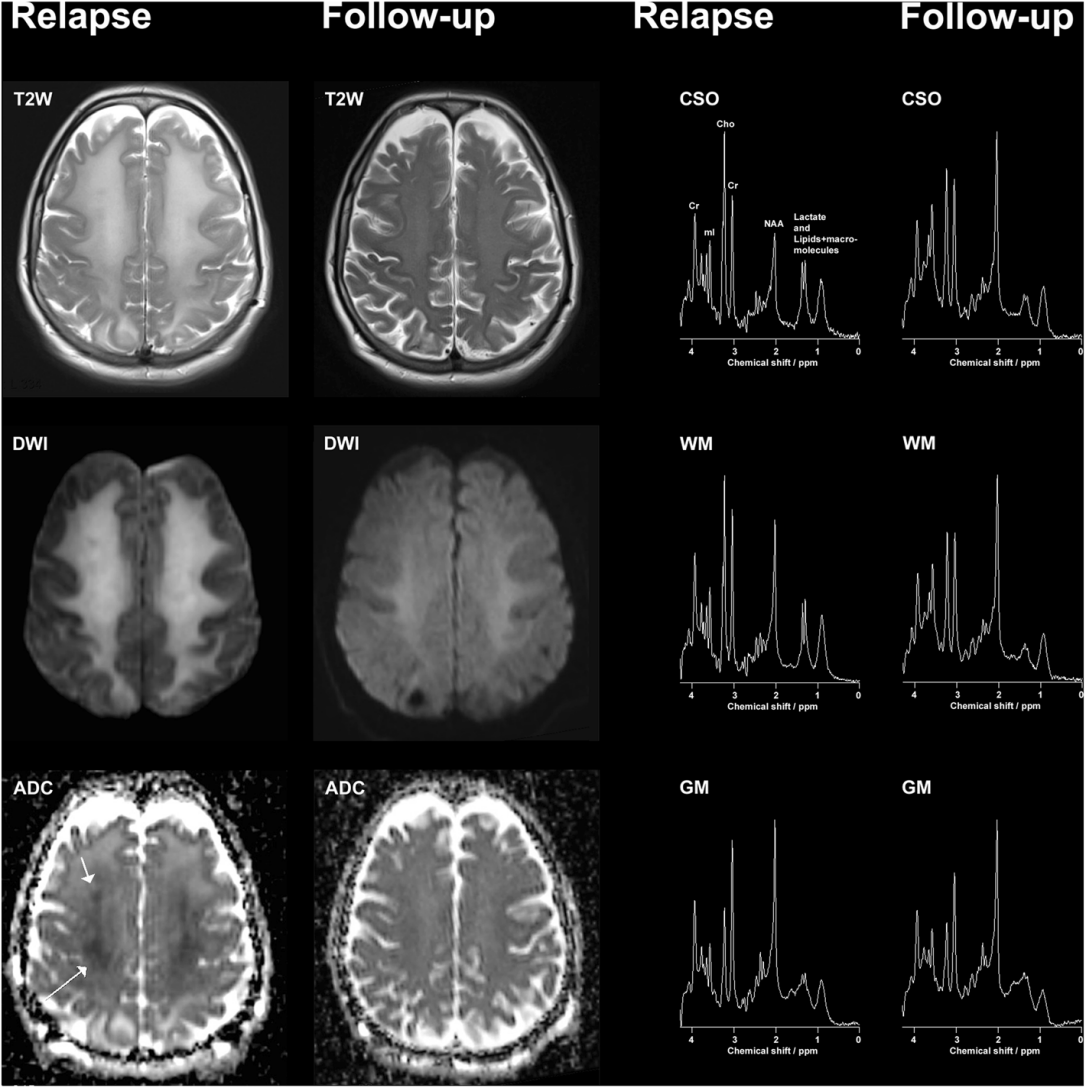
**Magnetic resonance examinations measured at 3 Tesla after relapse and at six-month follow-up.** T2W, T2-weighted magnetic resonance imaging; DWI, diffusion-weighted magnetic resonance imaging; ADC, apparent diffusion coefficient. CSO, centrum semiovale; WM, occipito-parietal white matter; GM, mid-occipital gray matter; T2-weighted magnetic resonance imaging, diffusion-weighted magnetic resonance imaging, and apparent diffusion coefficient images are displayed along with short echo-time magnetic resonance spectroscopy measured from centrum semiovale, occipito-parietal white matter, and mid-occipital gray matter.

A previously healthy 40-year-old Caucasian woman, a cigarette smoker, was found unconscious in a smoke-filled room near an open fireplace. She was lying next to her two dogs, one dead and one dying. The exposure time was unknown. Upon arrival of the Emergency Medical Services, our patient had a generalized seizure. She was admitted to an intensive care unit at the nearest hospital in a comatose state, Glasgow Coma Score 3. Arterial blood gas sample analysis revealed metabolic acidosis and hypoxemia with pH 7.29. Her carboxyhemoglobin (COHb) level was 22.6 percent. Serum biochemistry and complete blood counts were unremarkable except for leukocytosis (18.9×10^9^/L). Her initial brain computed tomography (CT) scan and lumbar puncture were normal. Due to her severe neurological symptoms, our patient was intubated and transferred to a tertiary hospital where HBOT was initiated in a multiplace hyperbaric chamber (Drass Galeazzi Underwater Technology, Livorno, Italy) within six hours after exposure to CO. Following five HBOT sessions (90 minutes exposure to 2.8 atmospheres absolute, (ATA)) breathing 100 percent oxygen supported by a ventilator (Siaretron 1000 IPERTM; Siare Engineering International, Bologna, Italy), our patient made a full recovery. She was discharged three days after admission. However, 28 days after discharge she experienced headache and a few hours later she was found in a confused state, standing in the snow without shoes.On admission to the hospital, an examination revealed akinetic mutism, muscular rigidity, and urinary incontinence. A CT scan of her brain showed diffuse low-density areas in the supratentorial white matter and these findings were confirmed in a subsequent MRI scan showing increased T2 signal diffusely in the white matter spreading through the internal capsule (Figure 1). The globus pallidus signal was normal, while the T2 signal of the putamen was slightly elevated. An acute bleeding of 12mm was present in the subcortical area of the right parietal lobe. Diffusion-weighted images showed diffusely increased signal in white matter, but the apparent diffusion coefficient was only decreased in smaller patchy areas in centrum semiovale.

Short echo-time MRS measured at 3 Tesla in occipito-parietal white matter (WM), mid-occipital gray matter (GM), and centrum semiovale (CSO) revealed severely increased lactate, lipids, and macromolecules. Additionally, a dramatic decrease in N-acetylaspartate (NAA) to total creatine (Cr) ratios was observed. Total choline (Cho) to Cr ratios and myo-inositol (mI) to Cr ratios were increased in CSO and mI/Cr was increased in WM (Table 1). The electroencephalography showed decreased background activity without paroxystical or focal episodes. The findings suggested severe leukoencephalopathy. Our patient received HBOT for the following six days, including three treatment sessions of 90 minutes at 2.8 ATA breathing 100 percent oxygen supported by a ventilator followed by three sessions of 90 minutes at 2.4 ATA breathing 100 percent oxygen with the use of a hood. During the course of HBOT, her clinical condition only improved minimally, and our patient suffered from severe impairment in concentration, verbal fluency, and executive functions. Furthermore, our patient appeared hypomimic. Due to the neurological sequelae, our patient was discharged to a neurorehabilitation center.

**Table 1:** Magnetic resonance spectroscopy in our patient at relapse (28 days post insult) and at follow-up (217 days post insult) compared to normal control values

	**NAA/Cr**	**Cho/Cr**	**mI/Cr**	**Lac/Cr**
**WM relapse**	−49% (−6.9 SD)	ns^***^	+21% (+2.6 SD)	+698% (+12.4SD)
**WM follow-up**	−17% (−2.3 SD)	+22% (+2.3 SD)	+38% (+4.6 SD)	ns
**GM relapse**	−37% (−4.4 SD)	ns	ns	+141% (+3.5 SD)
**GM follow-up**	ns	ns	ns	ns
CSO^*^relapse	−59% (−7.7 SD)	+73% (+7.8 SD)	+77% (+9.4 SD)	+918% (+16.3 SD)
CSO^*^follow-up	−29% (−3.8 SD)	+40% (+4.2 SD)	+55% (+6.8 SD)	ns^**^

WM, occipito-parietal white matter; GM, mid-occipital gray matter; CSO, centrum semiovale; NAA, N-acetylaspartate; Cr, total creatine; Cho total choline; mI myo-inositol; Lac, lactate; ns, not significant; SD, standard deviation. The metabolite ratios were calculated and compared to results in healthy subjects. The values reported are listed as deviations in percent from normal along with the corresponding SDs. The values were obtained using LCModel, and only values with Cramer-Rao lower bound (CRLB) less than 20 percent were considered. When CRLB was larger than 20 percent or the ratios were less than ±2 SD from normal, ‘ns’ was noted. The GM and WM ratios were compared to values from the same volumes of interest in a group of 17 normal controls used in the clinic for adult MRS patients.

^*^Exact control values were not available for CSO. The results provided for CSO were calculated as the best approximation using the WM control values. Metabolite ratios in CSO and WM differ probably less than 10 percent, but by the same factor for the two MRS examinations before and after recovery, so the dramatic recovery overshadows this approximation; ^**^in CSO at follow-up Lac/Cr is assigned the value, ‘ns’, because CRLB was 21 percent, if included the Lac/Cr ratio is 172 percent increased corresponding to 3 SD consistent with the visual detection of Lac in Figure 1; ^***^the Cho/Cr in WM at relapse is elevated based on a visual inspection, but the analytical evaluation based on the LCModel fit resulted in only 11 percent (+1.1 SD) elevated Cho/Cr: not significant. An inspection of the LCModel fit reveals that a shoulder of the left-hand side of Cho is not well accounted for by the fit. The LCModel fit accounts for glycerophosphocholine and phosphocholine. In this case yet another Cho-containing compound with a slightly different chemical shift may have been present at relapse causing the poorer fit of total choline. A similar shoulder was seen in the WM of CSO at relapse.

At the six-month follow-up, cognition was significantly improved; our patient was awake, alert and oriented. Her motor, sensory and cerebellar function were normal. An MRI scan showed notable improvement, but the T2 signal in WM was still slightly elevated. MRS showed extraordinary improvement (Figure 1). In GM, MRS had reverted to normal, consistent with neuronal recovery. In WM and CSO, NAA/Cr had almost returned to normal, lactate (Lac) was no longer significantly detected (Table 1), and mI/Cr had further increased, consistent with chronic gliosis. Our patient was independent in her activities of daily living, but complained about fatigability and impairment of memory and concentration.

## Discussion

The unusual and very remarkable findings in this case were the reversibility patterns of the severely reduced NAA/Cr and elevated Lac in GM and partial reversal in WM. The reversal of MRS abnormalities, and in particular the full reversal to normal in GM, has to our knowledge never been described before. Likewise, MRI improved substantially as may be seen after toxic encephalopathies [10].

Decrease in NAA reflects degeneration of neurons and axons or temporary dysfunction including mitochondrial dysfunction. The role of NAA is, however, yet not fully understood, as reflected by the total absence of NAA in a moderately well-functioning child with mental retardation [11]. Lac is elevated under conditions of ischemia or hypoxia due to anerobic glycolysis, but also during mitochondrial dysfunction. An increase in Cho indicates degradation of all cell membranes such as demyelination [12] or altered cell membrane turnover. In this case, the abnormal levels of NAA/Cr, Lac and Cho/Cr suggest disturbances in neuronal functioning, membrane metabolism and anerobic metabolism. Early decreased NAA/Cr and increased Cho/Cr as well as increased Lac are usually considered to be negative prognostic signs [6]. The patient in this case, however, regained good neurological function despite the initial severe symptoms and MRS measurements. Therefore, these results stand in contrast to other knowledge in this area.

CO-mediated encephalopathy is associated with diffuse demyelination of the cerebral WM [6]. However, the precise pathophysiology remains unclear. A possible explanation may be oxidative stress leading to inflammatory cascades but the neuropathological sequelae cannot solely be explained by hypoxic stress [13]. Thom *et al.* demonstrated that CO-mediated encephalopathy was linked to an adaptive immunological response to structural myelin basic protein in the rat brain [14]. An element of the mechanism may be mitochondrial dysfunction, which may be reversible upon recovery. The severely elevated Lac that had disappeared at the follow-up supports such a hypothesis.

Some of the mechanisms responsible for recovery may be explained by the potential benefits of HBOT when administered in the acute phase of intoxication [3]. A double-blind, randomized trial of HBOT on CO-mediated encephalopathy found that three HBOT sessions within a 24-hour period reduced the risk of cognitive sequelae six weeks and 12 months after acute CO poisoning, compared to breathing normobaric oxygen [3]. However, it is unclear whether HBOT improves outcome in CO-intoxicated patients when administered after the onset of delayed encephalopathy. Only two studies have addressed this concern, both of which found the treatment had a positive effect [15,16]. If HBOT has an effect on the resolution of the severe, diffuse leukoencephalopathy, then other patients with similar MRS characteristics might benefit from treatment, that is, drug-induced or other toxic leukoencephalopathy. This may give a new aspect to HBOT. In this case, we cannot know whether HBOT contributed to the final improvements in patient outcome or whether it was simply due to spontaneous evolution, as sometimes seen in other serious neurological conditions. Nevertheless, the condition of our patient did not deteriorate after the second round of HBOT sessions were initiated, which is important information to pass on to clinicians since literature in this area is lacking.

Molecular medicine in neuroscience has contributed important knowledge to the pathophysiology and biochemistry of neurological diseases and is a rapidly evolving field [17], especially in areas such as brain tumors, neurodegenerative diseases and neuroplasticity [18,19]. Positron-emission tomography and other molecular imaging methods might have added valuable information to our patient’s disease manifestation, disease course, and identification of treatment success in this case that could not be explained by the MRS. Future studies with larger study populations are needed in order to manage treatment based on MRS results and other molecular imaging methods should also be considered to be performed in disorders like this. However, this case provides valuable information regarding MRS results that can be used for future research in this area.

## Conclusions

This case suggests that even though MRS provides valuable information on brain metabolism in patients with delayed encephalopathy due to acute CO intoxication the prediction of long-term outcome is still complicated and even patients with severe, initial MRS measurements may show remarkable reversibility.

## Consent

Written informed consent was obtained from the patient for publication of this case report and any accompanying images. A copy of the written consent is available for review by the Editor-in-Chief of this journal.

## Abbreviations

ATA: atmospheres absolute; Cho: total choline; CO: carbon monoxide; Cr: total creatine; CSO: centrum semiovale; CT: computed tomography; GM: mid-occipital gray matter; HBOT: hyperbaric oxygen therapy; Lac: lactate; mI: myo-inositol; MRI: magnetic resonance imaging; MRS: magnetic resonance spectroscopy; NAA: N-acetylaspartate; WM: occipito-parietal white matter.

## Competing interests

The authors declare that they have no competing interests.

## Authors’ contributions

MBH contributed to the acquisition of data, drafting of the manuscript including writing and editing, data interpretation and drafting of the table. DK contributed to the drafting of the manuscript including critical revision of the manuscript for important intellectual content. ERD contributed to the acquisition of data, drafting of the manuscript, interpretation of the magnetic resonance spectroscopy and magnetic resonance imaging results, drafting of the figure and table and critical revision of the manuscript. VAL contributed to the drafting of the manuscript, interpretation of the magnetic resonance imaging results, drafting of the figure and critical revision of the manuscript. ECJ contributed to the acquisition of data and drafting of the manuscript including critical revision. OH contributed to the acquisition of data, data interpretation and drafting of the manuscript including critical revision. All authors have read and approved the final manuscript in its present form.
